# Assessment of Residual Radioactivity by a Comprehensive Wireless, Wearable Device in Thyroid Cancer Patients Undergoing Radionuclide Therapy and Comparison With the Results of a Home Device: A Feasibility Study

**DOI:** 10.1109/JTEHM.2020.3042118

**Published:** 2020-12-02

**Authors:** R. Gallicchio, D. Scapicchio, A. Nardelli, T. Pellegrino, M. Prisco, P. Mainenti, C. Sirignano, P. Pedicini, G. Storto

**Affiliations:** 1 Medicina NucleareIstituto di Ricovero e Cura a Carattere Scientifico (IRCCS), Centro di Riferimento Oncologico della Basilicata (CROB)85028Rionero in VultureItaly; 2 Istituto di Biostrutture e Bioimmagini, Consiglio Nazionale delle Ricerche 366199 80145NapoliItaly

**Keywords:** Wireless wearable device, permanent environmental home device, thyroid cancer, residual radioactivity, radiation exposure

## Abstract

**Objective**: To investigate the feasibility of using a wireless wearable device (WD) in differentiated thyroid cancer (DTC) patients undergoing radionuclide therapy with I-131 (RAI) and protected hospitalization, this study compared the measurements of residual radioactivity obtained with those registered by a permanent environmental home device (HD). **Methods**: Twenty consecutive patients undergoing RAI hospitalized in restricted, controlled areas were enrolled. The patients underwent comprehensive monitoring of vital/nonvital parameters. We obtained 45580± 13 measurements from the WD, detecting the residual radioactivity for each patient during approximately 56 hours of hospitalization, collecting data 53 times per hour. The samples, collected during daily activities, were averaged every two hours, and the results correlated with those from the HD. Bland-Altman analysis was also used to evaluate the agreement between the two techniques. **Results**: A significant relationship between the WD and HD was observed (r = 0.96, p < 0.0001). Bland-Altman analysis recognized the agreement between measurements by the WD and HD. The mean value at the end of the first day of hospitalization was 80.81 microSv/h and 60.77 microSv/h (p = ns for WD and HD), whereas those at the end of the second day were 47.08 and 24.96 (p = ns). In the generalized linear model (GLM), a similar trend in performance across time was found with the two techniques. **Conclusion**: This study demonstrates good agreement between the residual radioactivity measures estimated by the WD and HD modalities, rendering them interchangeable. This approach will allow both the optimization of medical staff exposure and safer patient discharge. **Abbreviations**: wireless device (WD); differentiated thyroid cancer (DTC); radionuclide therapy with I-131 (RAI); home device (HD); generalized linear model (GLM).

***Clinical and Translational Impact Statement***: This study demonstrates that a new wireless device can be used for patient radioactivity monitoring to allow both the optimization of medical staff exposure and safer patient discharge. Category: clinical implementation.

***Reprint requests and correspondence:** Mr. Giovanni Storto, Nuclear Medicine, Istituto di Ricovero e Cura a Carattere Scientifico (IRCCS), Centro di Riferimento Onco- logico della Basilicata (CROB), Via P. Pio 1, 85028 Rionero in Vulture, Italy Phone: C39 0972-726560 - Fax: C39 0972-726399 - E-mail: giosto24@hotmail.com Short Title*: Assessment of residual radioactivity by a wireless device.

Type of manuscript: original article

## I. Introduction

Dealing with patients undergoing therapy with radionuclides represents an operational challenge due to both historical misapprehensions and recent legal constraints [1]–[5]. In recent times, the correct management of such issues and the organization of the professionals involved has become a “must” in nuclear medicine departments [6].

Patients confined in restricted areas during hospitalization need frequent, operator-mediated monitoring of their vital/nonvital parameters, including radioactivity. Usually, environmental tools are implemented in nuclear medicine departments for the detection of patients’ residual radioactivity, providing fixed, temporal measurements that return only an estimate of the interpolated discharge curve [7].

The use of a mobile, wearable device during daily patient activities would provide instantaneous samples of the emitted radiation as well as repeated and accurate measures continuously. This approach would also allow both the optimization of professionals’ exposure and better sampling accuracy, which translates to safer patient discharge [8]–[10]. There is technological evidence for implementing wireless devices (WDs) in protected, controlled areas with restricted access by means of portable tools easily worn by patients, which constitutes an opportunity to support the appropriate management of nuclear medicine units. This technology has already been implemented successfully for monitoring cardiology patients during daily activities and may allow the simultaneous monitoring of multiple clinical parameters and residual radiation in a single modular device.

The implementation of the abovementioned system could conform to the current rules regarding job and environmental safety, and it will improve the procedures for reducing radiation and environmental exposure due to medical radioactivity [11]. The continuous monitoring of hospitalized patients in restricted areas might also enhance the daily patient/professional relationship to offer a more familiar approach. Overall, it constitutes an exportable model.

The aim of this study was to investigate the feasibility of using a newly constructed WD to estimate residual radioactivity and whether the measures are comparable with those obtained by a conventional environmental home device (HD). Contextually, we intended to implement a radioactive-patient management system to reduce working exposure while respecting the dignity of patients during solitary confinement in controlled areas. A significant reduction in the dose absorbed by professionals is expected.

## II. Materials and Methods

### A. Patients

**FIGURE 1.**
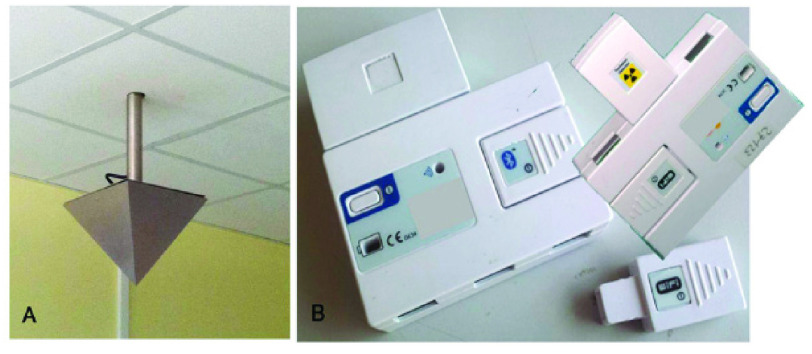
Image of the environmental home device (A) and of the wearable wireless device (B).

From October 2013 to January 2014, this study included 20 consecutive patients (8 men and 12 women; mean age, 46 ± {9} years) undergoing radionuclide therapy with I-131 (RAI) who were hospitalized in restricted areas. The administered activity range was 3626–4440 MBq. During the hospitalization days, all patients were monitored continuously for residual radioactivity by the HD and the worn WD at the same time (Figure 1).

Patients were carefully instructed regarding proper management of the wearable device. It consists of a tool for the comprehensive assessment of vital/nonvital parameters, such as blood pressure, cardiac frequency, pulse oximetry, body skin temperature, and body motion. Apart from the abovementioned clinical evaluation, the appraisal was focused on the residual radioactivity emitted. All patients who underwent RAI signed an informed consent form in accordance with the Declaration of Helsinki.

### B. Measurement Modalities

#### 1) Permanent Environmental Home Device

For the fixed, temporal measurements, we used a PC-based activity-measurement system to monitor the patient, the PADOS system (MED Nuklear-Medizintechnik, Dresden GmbH). This modality provides periodic measurements and the current radioactivity and/or the dose rate from the patient at rest.

Sample collection was performed by means of a collimated detector fixed on the ceiling of each protected room (2 m). The device was made of a scintillator detector (NaI) integrated in a shield, with an adapted collimator that defined a rectangular field of view over the patient’s bed. Data were analyzed, inverse square law-corrected, and stored by the PC system. An alarm advised the patient when the measurement was going to start and stop. Based on the periodically registered data, the system software was settled to calculate the expected moment of decreased radiation. This time represented the limit for safe patient release.

Using the PADOS system, we were able to obtain measurements at different times by four different sampling methods. First, a manual measurement of all patients was obtained after monitoring was ordered; second, a manual measurement of a single patient could be obtained; third, an automatic measurement of all patients could be obtained, preferably at night; and finally, a measurement with the device was obtained to measure the external dose rate. For each patient, the PADOS system acquired data every hour for 2,5 days, and then the collected data were averaged every two hours.

#### 2) Wireless, Wearable Device

To obtain instantaneous samples of the emitted radiation as well as repeated and accurate measures continuously, we implemented the RD2007, a high-performance X-ray detector that combines an Si-PIN photodiode preamplifier and a pulse discriminator in one device. This detector provides high sensitivity for assessing low-energy X-ray, beta, and gamma radiation with specific calibrated sensitivity for gamma radiation and inverse square law-correction of the data. In particular, this portable device was calibrated using a comparison technique against a reference standard that is periodically verified and traceable. The standard used was that of the Paul Scherrer Institute (PSI). The estimated uncertainty of this calibration was ±5% with respect to the reference standard.

This technology coupled with dedicated software was useful for monitoring both multiple clinical parameters and the residual radiation via a single modular device. Similar to the HD, for each patient, the WD acquired data 53 times per hour; then, the samples collected during daily activities were averaged every two hours, and the findings were correlated with those from the HD.

Additionally, in a subset of patients (#5), we compared the data from the WD with those obtained from the same device placed 1 meter away (fixed and timely registration).

**FIGURE 2.**
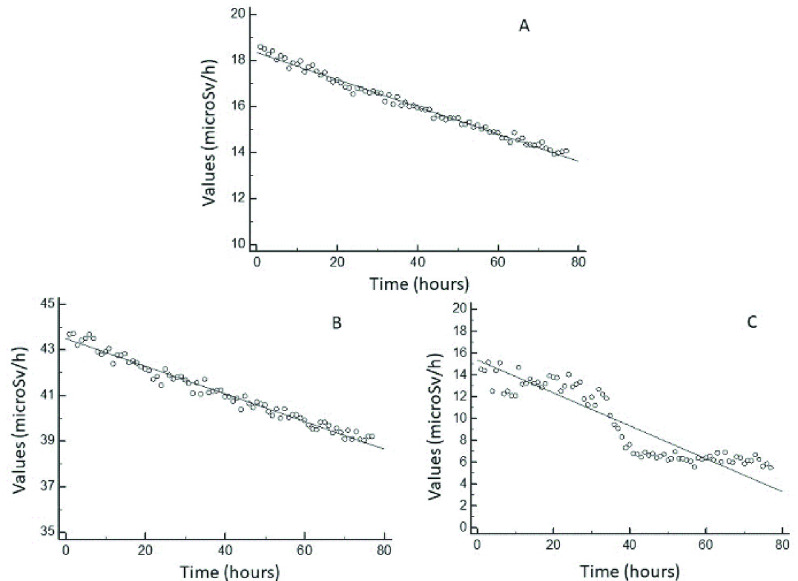
Linearity with the dose and dose rate of 654-MBq I-131 test-source activity for the environmental home device (A) and the wearable wireless device positioned near (B) and two meters away (C) from the source, respectively.

Linearity of the results with the dose and dose rate was tested using 654-MBq I-131 test-source activity for the environmental home device as well as the wearable wireless device positioned near and two meters away from the source, respectively (Figure 2).

This comprehensive monitoring system has also been tested for its ability to reduce radiation exposure for workers (#8) compared to current procedures (TLD and electronic staff dosimeters). Monthly dose rates were compared to historical data verifying whether the adopted system ultimately optimizes the working procedures.

## III. Statistical Analysis

Continuous data are expressed as percentages, the mean ± SD, and the median, as appropriate. Comparisons between the mean values were performed with paired and/or unpaired Student’s t test (two-tailed probability), when necessary. Correlation analysis was used to assess the relationship between samples collected by the WD and HD systems. Bland-Altman analysis was used to evaluate the agreement between the two techniques [12]. With this method, the differences between the two modalities were plotted against the mean data from the WD and the HD. If the limits of agreement (mean ± 1.96 times the SD of the differences) were not clinically important, the two methods were considered interchangeable. The statistical relevance of differences during the monitoring period between the two methods (HD and WD) was considered. To compare the data, a generalized linear model (GLM) for repeated measurements (two-way ANOVA) was employed. With this method, we assessed for each patient, under the experimental conditions, whether there was a relationship between the WD and HD. The data were averaged. A post hoc analysis with Bonferroni’s correction was performed when appropriate. A $p$ value < 0.05 was considered statistically significant.

## IV. Results

### A. Patients

Data from all the enrolled patients were available, and no technical issues were encountered during the monitoring period. They easily wore the device without noncompliance concerns in a contest of overall wellbeing. Patient data were continuously transferred despite a lead-protected environment. Incidentally, most patients (17/20; 85%) revealed an unexpectedly high rate of long-lasting hypothermia (32°C ± 3) and bradycardia (45 beats/min ± 15) that can be present in this condition (hypothyroidism) but is not usually demonstrated.

### B. Measures from the Home Device and Wireless Device

For each patient, the PADOS system acquired data for 56 hours, and then the collected data were averaged to obtain 28 measures (every two hours). Overall, 45580± 13 measurements from the worn WD were obtained, detecting the residual radioactivity for each patient during approximately 56 hours of hospitalization and collecting data 53 times per hour. Data were averaged every two hours, resulting in 28 measures.

**FIGURE 3.**
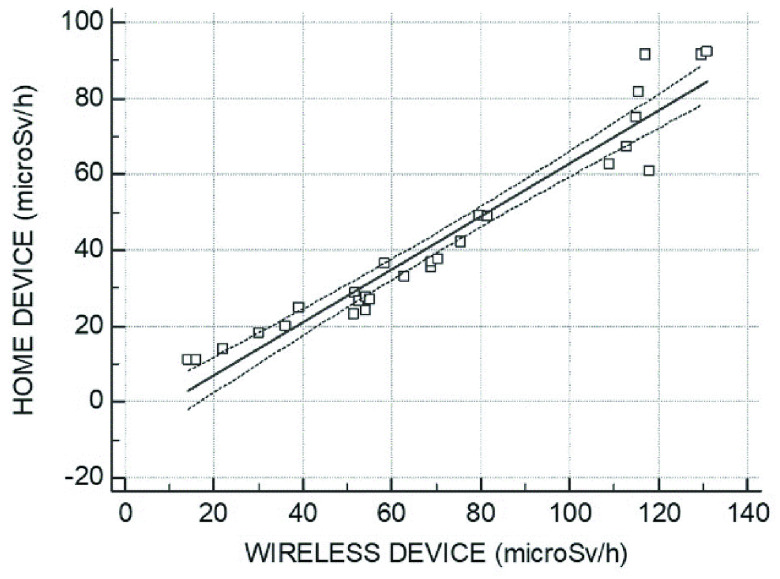
Relationship between the residual radioactivity measured by the home device and wireless device. The *solid line* indicates the regression line, and the *dashed lines* indicate the 95% confidence interval.

**FIGURE 4.**
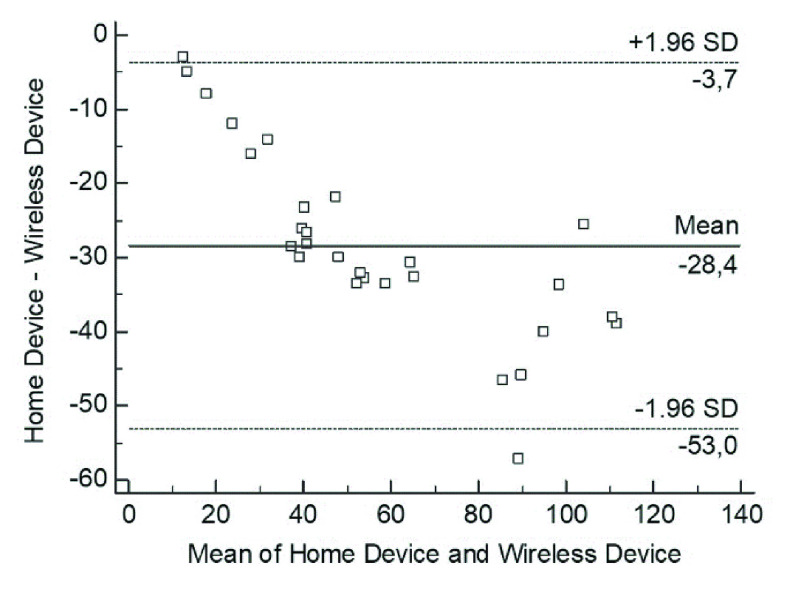
Bland-Altman analysis. Agreement between the residual radioactivity measured by the home device and wireless device. The differences between the two methods are plotted against the means of the two tools. The *horizontal solid line* indicates the mean difference between the two methods, and the *dashed lines* indicate the limits of agreement (mean difference ± 1.96 times the SD of the difference).

The mean values at the end of the first day of hospitalization were 80.81 microSv/h and 60.77 microSv/h for the WD and HD, respectively (p = ns), whereas those at the end of the second day were 47.08 microSv/h and 24.96 microSv/h (p = ns). A significant relationship between the WD and HD measures was observed (r = 0.96, p < 0.0001) (Figure 3). The mean difference between the residual radioactivity measured by the HD and WD was −28.36, and the SD of differences was 12.58. The lower and upper limits of agreement between the two modalities were −53.03 and −3.69, respectively (Figure 4). Finally, Bland-Altman analysis confirmed the agreement between the measurements obtained by the WD and HD.

**FIGURE 5.**
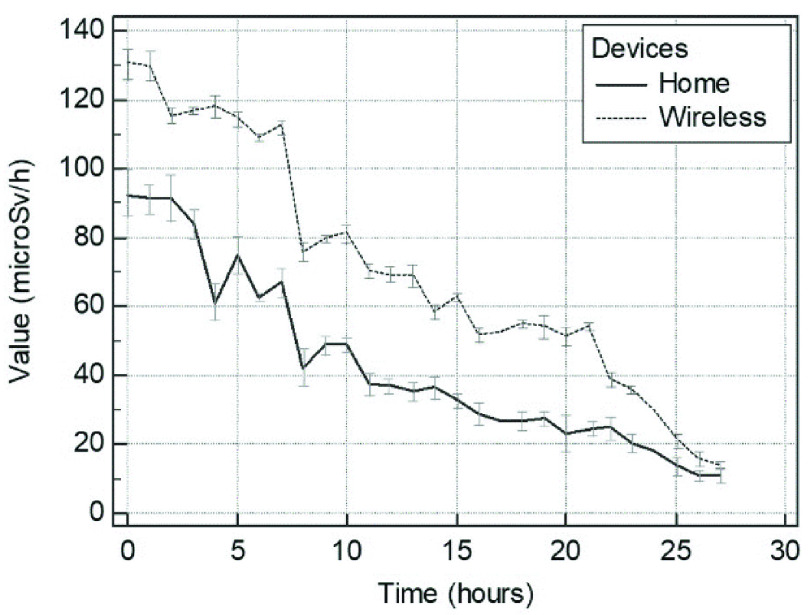
Reduction of the residual radioactivity of patients detected over time by the home device (*solid line*) and wireless device (*dashed line*). Monitoring was performed over 56 hrs of hospitalization with measures averaged every two hours. Uncertainties are displayed.

The GLM indicated significant differences in the absolute value of measures between the two techniques over time (Figure 5). Among the other time points, the representative mean values at baseline (time 0), 12 h (time 6) and 36 h (time 18) were 131.09 microSv/h, 109.01 microSv/h and 55.04 microSv/h, respectively, for the WD and 92.09 microSv/h, 62.45 microSv/h and 26.77 microSv/h, respectively, for the HD (all $p < 0.001$). Despite this, a similar trend was displayed, and the values at discharge were 14 microSv/h and 11 microSv/h, respectively ($p =$ ns). With both methods, a significant reduction in the detected radiation occurred at 16 h (time 8), when patients probably discharged the most considerable amount of radioactivity (Figure 5).

The mean values obtained immediately after activity administration, at the end of day 1, and at the end of day 2 (#5 pts) were 148.4 microSv/h, 102.6 microSv/h and 42.1 microSv/h, respectively, for the worn WD and 0.10 microSv/h, 0.07 microSv/h and 0.03 microSv/h, respectively, for the device positioned 1 meter away (all $p < 0.001$), which approximates the expected theoretical values.

### C. Laboratory Calibration, Linearity, and Half-Life

The calibration of the home system was performed using a 1150-MBq I-131 test source positioned at a 2-meter distance (reference distance). By comparing this value to the measured value, the calibration factor was calculated. Through the calibration factor (CF), the count rate measured by the probe was converted to the dose rate. The CF indirectly indicates the dose rate constant (0.059 microSv m2/h/MBq). Before starting, the wireless device was calibrated using a Cs-137 (0.037-MBq) radiation source under the following measurement conditions: radiation equivalent dose rate, 2.2 microSv/h; measurement time, $\ge {8}$ h; supply voltage, 5.00 V; and ambient temperature, $22~^{\circ }\text{C}~\pm 2$. According to the abovementioned conditions, 2.8 counts per minute for a 1-microSv/h radiation equivalent dose rate was obtained. Linearity with the dose and dose rate, tested using I-131 test-source activity, is shown in figure 2. The two detectors appeared to respond similarly, with the same trend. Nevertheless, when the WD was positioned at two meters, the measures were unreliable (Figure 2C), according to the rationale that the device was designed to be worn. Concerning the effective half-life resulting from testing the HD and WD, the slopes were 0.0590 and 0.0598, respectively. As a measure of the trend, the linear equations were similar (y $= 18.3+ -0.0590\text{x}$ vs y $= 43.4+ -0.0598\text{x}$; residual SD: 0.151 vs 0.223).

### D. Radiation Exposure

The monthly mean dose rates for the 8 workers involved were 0.8 ± 0.2 mSv compared to 1.2 ± 0.6 mSv, the latter of which represents the consolidated historical datum (p < 0.001).

## V. Discussion

Radiation protection relies on meeting requirements that apply three principles adopted in most regulatory systems throughout the world: justification, optimization, and limitation of individual doses. It is mandatory to respect the limit of doses that various groups may receive, including workers and the general public. The issue arises when patients undergo radionuclide therapy and ought to be hospitalized in restricted, controlled areas [13]. In addition, it should be considered that such therapy lies in the context of a public misconception essentially related to recent accidents and environmental radioactive events [14]. Increased public and legislative awareness is expected after a brief delay.

At the end of some therapeutic nuclear medicine procedures with unsealed radionuclides, safety measures are mandatory to limit doses to other people (the public), while this is rarely the case after diagnostic procedures. Iodine-131 results in the largest dose to medical staff, public caregivers, and relatives [15]. Basically, dose limits are related to the exposure of the public and medical staff from patients [16]. The modes of exposure for professionals involved include external exposure, internal exposure due to contamination, and those correlated to workplace pathways [11], [17]. Accordingly, the decision to hospitalize and release a patient must be determined on an individual basis considering many factors [18].

Environmental or other radiation-detection devices can be used to monitor patients who have received radioiodine therapy during hospitalization and for several weeks after treatment. Records from the specifics of therapy with unsealed radionuclides are kept at the hospital and used to assign precautionary instructions to patients, along with written consent.

Wireless technology sustained by portable devices, easily worn by patients, constitutes an opportunity to achieve the appropriate management of nuclear medicine units. Vital/nonvital parameters and residual radioactivity during daily activities can be continuously registered [19]–[21].

Our study was undertaken to implement a wireless wearable device in protected areas and to validate its performance on a daily basis compared to a permanent environmental home device. The feasibility of such an approach is encouraging since measures obtained from the wireless device correlate with those acquired by the validated, fixed system. As a result, the device could easily be implemented in routine daily activities because it is interchangeable with conventional existing tools. It appears that protected hospitalized patients could be managed easily in terms of both professionals’ safety and patients’ control. At this time, there is technological evidence for implementing wireless devices in protected, controlled areas with restricted access to reduce the possibility of undue radiation exposure to professionals [22]. From a technical point of view, it allows more detailed and precise sampling, which translates to more interpolated decay curves at the time of patient discharge.

At the same time, this technology could improve patient-physician relationships and checks [23]. In fact, it is well known that patients referred to a nuclear medicine department (therapy) have several anxiety-predisposing factors (e.g., hypothyroidism, cancer-related apprehension, confined hospitalization-related uneasiness) [18]. During monitoring, the patients appeared to have an unexpected high rate of long-lasting hypothermia and bradycardia that could be expected in hypothyroidism [24] but has never been demonstrated before. Ultimately, this new wireless technology allows a comprehensive approach to patient management [25], [26]. From an organizational point of view, our data confirm the hypothesis of better global monitoring of patients forced to be hospitalized in protected areas because of their radioactivity. The automation of processes for measuring physiological parameters, a reduction in medical staff exposure to ionizing radiation and a diminution of clinical risk are expected.

Data on measures showed a strong relationship between the two different devices used. The Bland–Altman analysis supported the interchangeability of the fixed and portable tools, which translates to good agreement and repeatability of the measures. As a result, the wearable device appears to be validated and could be used efficiently.

The GLM confirmed the performance over time, which is crucial for this monitoring system. In fact, the decay curves from the two devices showed a similar trend. Both devices detected a significant decrease in the radiation emitted when patients began to discharge radioactivity because of their effective half-life. The home device exhibited higher time-related variability due to the normal dwelling of patients outside of bed. Conversely, the WD appeared to continuously detect a greater number of events, avoiding the previous issue. The measures were closer to each other at discharge, when the standard deviation was expected to have less of an impact due to the low values detected at this time, since the lower the measures are, the lower the inaccuracy. It can be disputed that the differences between the measures obtained by the two devices increased when the detected radiation increased (Figure 5). However, the most important cause could be related to the movements of patients who cannot be completely controlled. In fact, especially during the first part of hospitalization (when dose rate is higher), the patients are somewhat unable to stay at rest during measurements (resulting in fewer measures by the HD), which was not the case for the wireless device upon testing for linearity. The same issue might impact half-life appraisal.

Such technology coupled with dedicated software has already been implemented successfully for monitoring cardiologic patients during daily activities [27], [28]. On the other hand, the economics of these devices are continuously disputed. Several attempts have been made to determine the optimal location of the detector on the body as well as the optimal environment for a fixed device [7], [29], [30]. However, the implementation of our device is feasible, and it provides an exportable monitoring model that can be easily adopted in several hospitals (transferability). Apart from the intrinsic characteristics of the two devices and discrepancies due to physics (e.g., lab calibration and response of materials used), the two curves depicted in figure 5 are substantially similar (same trend) and consequently may allow the determination of a caliper factor granting the interchangeability of the two systems and ultimately their reciprocal replacement.

Prospectively, this monitoring approach could also be extended to other peculiar environments, such as MRI scanners and cyclotron facilities (by switching the sender and receiver; reversibility) and implemented for managing radioactive materials. The technology has features that support both healthcare and job safety policies as well as environmental security. Additionally, this technology could expand the methodologies used so far and allow us to adhere strictly and easily to policies on radioactive residuals from medical activities, which are expected to become stricter in upcoming years.

**Limitations**

The results of our work should be interpreted in view of certain limitations. First, the data were not validated against a globally recognized reference method (see standards and calibration), which limits the precision of the absolute values measured. While this was not the purpose of the present study, it will be the topic of a future investigation. Concerning the measures, technical hurdles and some methodological differences between the two compared devices could occur, especially when dealing with the compensation for different counting geometries, but feasibility was proven.

## VI. Conclusion

The results of this study indicate that the use of a wireless, wearable device to monitor the residual radioactivity of patients who are hospitalized for radionuclide therapy in restricted, controlled areas is feasible and readily available, with performance as accurate as that of conventional fixed devices. Additionally, when used in a comprehensive modality (vital, nonvital parameters), it reduces and optimizes the contact between operators and patients during daily activities and may constitute added value in the medical radiation framework.
